# The Mental Treatment Bill

**Published:** 1923-06

**Authors:** 


### THE MENTAL TREATMENT BILL.

### INTERVIEW WITH SIR FREDERICK WILLIS.

THE Mental Treatment Bill, which has been read a second time in the House of Lords, contains provisions of far-reaching effect on the treatment of persons suffering from mental diseases and deserves the careful consideration of those who are interested in public health administration.

### The Need for Early Treatment.

Sir Frederick Willis, K.B.E., Chairman of the Board of Control, in an interview which he courteously granted to a representative of The Hospital and Health Review, explained that the amendments in the existing Lunacy Laws proposed in the new Bill had been desired by the Board for some considerable time, and had been approved by local authorities and by representative medical men who had specialised in the treatment of mental diseases. Probably the most important matter which had exercised the minds of those who were associated with the treatment of the mentally diseased was the need for encouraging expert treatment at an early stage. Medical science was increasingly making it possible to regard many forms of mental illness as being definitely curable; but experts were agreed that if the mentally disordered could be encouraged to apply for treatment at an earlier stage, the percentage of cures would be much higher. Under the existing laws it is difficult to provide early treatment without certifying the patient as insane under the Lunacy Acts. The well-known strong prejudice against certification too often results in the patient seeking expert advice only after the curable stage of the disease had been passed. The Bill encourages a mentally diseased person to seek early treatment in three ways: As a temporary uncertified patient in a public mental hospital; as a voluntary boarder in a similar institution; or as an out-patient.

### Treatment Without Certification.

The most important innovation in encouraging early treatment is contained in Section 4 of the Bill. This section provides, in effect, that a person suffering from mental disorder and likely to benefit from temporary treatment in an institution, may, without certification, be received in an approved institution for treatment for a period which may be extended up to one year. The initial recommendation for this treatment is made by two doctors, one of whom must have been approved for the purpose, either by the Board of Control or by the local authority. Where the patient has volition, this special procedure without certification will apply only where he is willing to submit to the treatment. If the patient is incapable of volition the procedure may be applied on the certificate of a Justice of the Peace or minister of religion personally acquainted with him. The uncertified patient is entitled to leave the institution at any time on giving forty-eight hours' notice—he cannot be detained against his wishes. He can be discharged under conditions similar to those which govern the discharge of private patients from lunatic asylums. The words defining the institutions in which this form of treatment may be carried out are made purposely wide to meet the varying conditions in different parts of the country, the aim being that early treatment should in all cases be encouraged and should not be withheld merely because of the absence in any area of some particular type of building or organisation. The Bill provides that "approved Institutions" shall include institutions under the control of a Visiting Committee (this means the public mental hospitals of the County and Town Councils); registered hospitals or licensed houses approved by the Board of Control; and any unregistered hospital or part of one approved by the Board of Control whose managers have concluded an agreement with the Visiting Committee of the County or County Borough Hospital or with the managers of a registered hospital.

### Wide Discretion of Visiting Committee.

The Bill specifically authorises Visiting Committees to enter into such agreements on such terms and conditions as they think fit. The elasticity of the last definition is noteworthy. Where it is possible to get a general hospital to work in conjunction with the Visiting Committee the expert facilities of the former can be used to great advantage in the early treatment of mental cases. On the other hand, where such ideal conditions do not exist, it will still be possible for some neighbouring villa or other suitable building to be used. It is desirable to emphasise that the proposals for early treatment without certification are entirely unconnected with the Poor Law—the patients will not, in any degree, be pauper patients. The local authority will arrange the financial terms on which treatment will be given and any cost falling on the authority will be charged to the authority's general funds and not to the Guardians of the Poor.

### The Voluntary Boarder.

Since 1890 registered hospitals and private asylums have been allowed to receive as voluntary boarders persons suffering from mental disease, but this right has been denied to the Visiting Committees of public mental hospitals. At the Conference convened in London last year to consider improvements in Lunacy Administration it was unanimously agreed that the extension of the system to County and County Borough mental hospitals was desirable, as this would enable the poorer sections of the population to take advantage of a system which had proved to be of considerable value in encouraging early treatment. The Bill provides briefly that Visiting Committees shall have power to receive and lodge as a boarder and to maintain and treat any person who desires voluntarily to submit himself to treatment. The Committee are given full power in making terms with the boarder and can thus vary the scale of charges to meet the circumstances of the individual patient. The boarder may leave the institution on giving forty-eight hours' notice.

### The Out-patient Treatment.

It had for long been felt that some system of out-patient treatment should be linked up with the work of the public mental hospitals. Hitherto Visiting Committees have had no authority to proceed in this direction; this disability it is proposed to remove by the Bill, which empowers such Committees to make provision, either gratuitously or on such terms as they think fit, for the out-patient treatment of persons suffering frcm mental disorder. The provision is in general terms so that the maximum advantage may be gained from local conditions. Frequently, no doubt, the out-patient department or clinic will be established in connection with the general hospital of the district, but the Visiting Committee will have a free hand to make any other arrangements.

### The Controlling Authorities.

In connection with the early treatment of mental disease, and particularly with the newly recognised principle of temporary institutional treatment without certification, there arises the very important question whether this treatment should be supervised and carried out by the central authority and the local bodies which are already dealing with the care of certified cases, or whether this early treatment should be controlled by a separate Committee of the local authority, supervised by some central department other than the Board of Control. The Bill recognises the former principle, a course which was strongly urged at the London Conference last year. There are several weighty reasons to support this policy. Financially, the setting-up of any dual system would be exceedingly costly—it would mean a duplication of medical, nursing and other staff, and would necessitate the provision of some thousands of new beds. From the administrative standpoint, too, a duplication of control would hardly tend to efficiency. The most important argument against dual control rests, however, on the definitely curative tendency in the treatment of mental diseases to-day. The progressive abandonment of the word "asylum" in favour of "hospital" represents a very real principle. The modern view is that the lunatic asylum should be, in real fact, a mental hospital whose main object is the cure of its patients, rather than their permanent incarceration. Not only are the mental hospital authorities imbued with this hospital spirit (it is significant that there has been no demand from them for dual control), but the medical staffs are strongly insisting on the extreme importance of the curative aspect of mental hospitals.

### The Hospital Spirit.

By associating the treatment of curable cases with the existing public institutions, a better feeling towards such hospitals will be encouraged in the minds of the mentally afflicted and of their relatives, and much will be done to remove that sentimental objection to entering a "madhouse," which hitherto has represented one of the main difficulties in encouraging application for early treatment. On the other hand, by withdrawing from the existing public mental hospitals the care and treatment of curable cases, such institutions will necessarily come to be regarded as prisons for the hopeless and the hospital spirit will languish. It will obviously be impossible either to retain or recruit the most capable medical men or nurses for service in such institutions. The principle adopted by the Bill is, therefore, to encourage treatment by local authorities, through one Committee, of all cases of mental illness, whether early or late, uncertified or certified, and thus to foster the hospital spirit in existing institutions under the best scientific conditions and to assist in removing the stigma which still attaches in many minds to the public lunatic asylum.

### After-Care.

Another important principle recognised by the Bill is the need for providing facilities for caring for patients after their discharge from an institution. Experience has shown that a patient will often re-establish himself after discharge if he is given suitable help in a congenial environment, although, in the absence of any such sympathetic assistance, there is danger of a renewed breakdown. The Bill is, very happily, elastic. The Visiting Committee is empowered, in general terms, to make provision for the care, after discharge, of any person who has undergone treatment in an institution for patients suffering from mental disorder. This is a branch of work where, in the opinion of the Board of Control, voluntary association and effort can be used to great advantage and should be encouraged in preference to any rigid official control. Already there exists the Mental After Care Association, and it would, for example, be possible, under the terms of the Bill, for local authorities to work in conjunction with such a voluntary association.

### Research.

The pressing need for extended research work in connection with the treatment of mental diseases is now generally recognised. The causes of many mental illnesses are still largely unknown. If a substantial advance is to be made in this branch of medical science it is essential that a more accurate knowledge of the causes of insanity should be obtained, and this can be done only by exhaustive research work. The Bill empowers Visiting Committees, with the consent of the local authority, to undertake research in relation to mental disorder and its treatment and to make contributions towards the expenses of any body of persons engaged in such research. While in most of its provisions the Bill aims at elasticity of application, it was felt that in research work some degree of co-ordination throughout the country was necessary to avoid waste of energy. The Bill, therefore, provides that the schemes of research proposed for adoption by the local authorities should be approved by the Board of Control. Sir Frederick Willis quoted the examination of fæces as an example where co-ordination was clearly desirable. If it were agreed that the fæces of a large number of lunatics should be examined in order to ascertain whether a particular type of bacillus was generally associated with a particular form of insanity, the value of the investigation would be seriously impaired, if not indeed destroyed, unless uniformity of practice were adopted.

